# Niche overlap and species co-occurrence patterns in carabid communities of the northern Chinese steppes

**DOI:** 10.3897/zookeys.1044.62478

**Published:** 2021-06-16

**Authors:** Noelline Tsafack, Xinpu Wang, Yingzhong Xie, Simone Fattorini

**Affiliations:** 1 School of Agriculture, Ningxia University, 489 Helanshan West Road, 750021, Yinchuan, Ningxia, China Ningxia University Yinchuan China; 2 C3EC – Centre for Ecology, Evolution and Environmental Changes/Azorean Biodiversity Group and Univ. dos Açores, Depto de Ciências e Engenharia do Ambiente, Angra do Heroísmo, Açores, Portugal Centre for Ecology, Evolution and Environmental Changes/Azorean Biodiversity Group Angra do Hero&iacute;smo, A&ccedil;ores Portugal; 3 Department of Life, Health and Environmental Sciences, University of L’Aquila, 67100, L’Aquila, Italy University of L’Aquila L'Aquila Italy

**Keywords:** Carabidae, community organization, competition, co-occurrence, c-score, niche overlap, niche segregation, null models

## Abstract

Understanding how species sort themselves into communities is essential to explain the mechanisms that maintain biodiversity. Important insights into potential mechanisms of coexistence may be obtained from observation of non-random patterns in community assembly. The spatial niche overlap (Pianka index) and co-occurrence (c-score) patterns in carabid species in three types of steppes (desert steppe, typical steppe, and meadow steppe) in China was investigated. Non randomness was tested using null models. Niche overlap values were significantly higher than expected by chance in the desert steppe, where vegetation cover is less abundant and less uniformly distributed, which possibly forces species to concentrate in certain places. In the typical and meadow steppes, results were influenced by the scale of the analysis. At a broad scale, niche separation was found as a result of species segregation among different sectors (habitats) within these steppes, but when the analysis was conducted at a finer scale, species appeared to be no more segregated than expected by chance. The high co-occurrence averages found in the meadow and typical steppes indicate that the distributions of the species found in a site may be negatively affected by the presence of other species, which suggests that some species tend to exclude (or reduce the abundance of) others. The very low c-score average observed in the desert steppe suggests that competition is not involved there. Thus, in more homogeneous landscapes (such as the typical and meadow steppes), competition might play some role in community structure, whereas spatial variation in the abundances of species is more driven by the uneven spatial distribution of vegetation in the landscape where productivity is lower and less uniformly distributed.

## Introduction

The niche concept formalized by Hutchinson (1957) is centered on species co-occurrence. The fundamental niche represents an n-dimensional hypervolume in which each dimension corresponds to a state of the environment which would permit a species to exist indefinitely in the absence of other species (Chase and Leibold 2003). While the fundamental niche is a purely theoretical concept because usually species do not use all the n-dimension in their environment, the realized niche is the part of the fundamental niche which is actually occupied by the species after its interactions with other species (Vandermeer 1972; Soberón and Arroyo-Peña 2017).

Niche overlap describes the situation in which co-occurring species share parts of their niche space with each other. High niche overlap may lead to conflictual interactions (such as competition and exclusion) for some species (Giménez Gómez et al. 2018; Pascual-Rico et al. 2020) or only indicate strong interactions in one niche dimension, with coexistence permitted by only weak interactions in others (Koutsidi et al. 2020). In contrast, low niche overlap generally indicates lower levels of interaction, thereby allowing sustainable co-existence of species (Koutsidi et al. 2020; Yang and Hui 2020).

Low niche overlap, which implies differential utilization of resources, is considered essential for the coexistence of syntopic species and hence to promote persistent diversity. In particular, interspecific overlap in habitat use has been invoked as a key factor in shaping ecological communities from the small scale (e.g., close-range interspecific interactions; Lombardo and Mjelde 2014) to the large scale (e.g., species distribution areas, including evolutionary aspects; Holt 2009).

The role of interspecific competition in determining insect community organization and diversity is highly debated (Hairston et al. 1960; Price et al. 2011; Schowalter 2011; Fattorini et al. 2020). A first cause of uncertainty is that obtaining direct evidence of competition through experiments (Price et al. 2011) is extremely difficult. However, an interesting alternative is to infer the possible presence of competition by testing whether observed patterns of resource utilization are statistically different from those expected based on chance. For example observed values can be compared with those obtained using null models, and the existence of competition inferred, assuming that competition will produce values of niche overlap lower than a random utilization of a certain resource (Gotelli and Graves 1996). Further sources of difficulty in assessing the role of competition in insect community structure flow from the large number of species of insects in a community and the many consequent factors affecting community structure. For these reasons, important insights into the role of niche partitioning in insect community structure may come from studying simple systems with small number of species (Fattorini et al. 2016).

In this paper, we investigated niche overlap in carabid beetle communities in Chinese steppes using null model approaches to disclose potential mechanisms of co-existence. In Chinese steppes, carabids are among the most abundant ground dwelling insects (Yu et al. 2004; Liu et al. 2015; Zhang et al. 2017), but the severity of the environment is associated with co-existence of few species (Tsafack et al. 2019a), and the question about their niche partitioning remains unsettled. Chinese steppes are classified into various types according to their main vegetation, which is in turn influenced by different climatic conditions, especially humidity. For our study, we considered three steppe types that reflect a gradient of aridity from the most arid to the most humid: desert steppe, typical steppe and meadow steppe (Kang et al. 2007).

Using intensive sampling by pitfall trapping in each type of steppe, we tested if patterns of species co-existence deviated from random as defined by using randomization processes that disrupt the original structure in the data according to more or less restrictive rules. For example, one can choose that places unoccupied by a given species in the field data are constrained to be also empty in the simulated null-assemblage, or one may adopt a more liberal approach, in which such sites may receive those species in the null-assemblages. Such null-assemblages generated by randomization represent what one can expect if no biological mechanisms regulate species coexistence. Significant deviation of observed patterns from assemblages predicted by the null-models provides evidence that some structure occurs and hence existence of some biological mechanism is implied.

We used null model approaches based on two different sets of rules to assess if spatial niche breadth and overlap patterns in the carabid communities of these steppes can be explained by chance alone, or if there is some spatial partitioning. Under the null-hypothesis that patterns are not structured by biological mechanisms (H0), i.e., if species abundances are randomly distributed within a given ecosystem, we expect no difference between observed and random values of niche overlap. This null-hypothesis can be falsified either by significantly higher (H1) or lower (H2) values of niche overlap compared to those obtained by random models. Niche overlap values higher than those expected under a null model in which species are allowed to occupy any of the places available, even those actually empty (but similar to those obtained for null-assemblages in which species were not allowed to occupy empty places), indicate that the presence of highly unsuitable places play a major role in community structure by forcing species to coexist. In contrast, niche overlap values lower than those expected under a null model in which the zero-structure is preserved (but similar to those obtained when the structure of zero values in the matrix is destroyed) are consistent with an inference that the community is structured by species segregation processes mediated more by species interactions than habitat heterogeneity. Based on the habitat structure of the three ecosystems, we predicted that H1 would be verified in the desert steppe, where resources are more fragmented (relatively isolated patches of vegetation which drive species coexistence), whereas H2 would be verified in the typical and meadow steppes (where vegetation is more uniformly distributed and hence niches are expected to be more influenced by species interactions).

## Materials and methods

### Study area and sampling design

The study was conducted in the Ningxia region (northern China), between 36°N and 38°N and between 105°E and 108°E, in three types of grassland ecosystems: desert, typical and meadow steppes (Kang et al. 2007). A map of the study area with photographs of the three grassland ecosystems is given in Tsafack et al. (2019b). The desert steppe was characterized by a vegetation mainly represented by drought-tolerant species such as *Agropyron mongolicum*, *Artemisia desertorum*, *Artemisia blepharolepi*, and *Stipa* spp. The typical steppe included natural patches of grass (*Stipa bungeana*, *S. grandis*, *Artemisia frigida*, *Thymus mongolicus*, *Heteropappus altaicus*, and *Potentilla acaulis*) in association with cut grasses used as fire belts and crops. Vegetation in the meadow steppe was mainly represented by *Festuca brachyphylla*, *S. bungeana*, *A. frigida*, and *Achnatherum splendens*.

We selected three main sampling areas representing these three types of steppes. To reflect within-ecosystem variability of the typical and meadow steppe habitats, on the basis of vegetation characteristics, we identified three habitat types (that we refer to as sectors 1, 2, and 3) within the typical steppe, and two habitat types (sectors 1 and 2) in meadow steppe. Data were gathered from 90 sampling sites distributed as follows: 15 sites in the desert steppe, 45 sites in the typical steppe (15 sites in each sector), and 30 sites in the meadow steppe (again 15 sites in each sector). In each sector, sites were selected haphazardly (i.e., without any regular spatial arrangement) and separated by at least 150 m to avoid, or at least reduce, possible autocorrelation.

At each sampling site, five pitfall traps (separated by at least five meters from each other) were installed. Pitfall traps consisted of 7.15 cm-diameter plastic cups, sunk in the ground with the cup-lip level with the soil surface, and filled with 60 ml of a mixture of tap water and vinegar (8%), sugar (4%), and 70% alcohol (4%). Sampling was done from May to September 2017. During the sampling period, pitfall traps were placed in the sites once a month in mid-month, and left in the field for 72 h prior to collection. Traps were composed of two buckets, with the smaller inserted into the larger. At each sampling session, the smallest were extracted to collect the trapped beetles and then paced again in the largest, which were left dug into the soil. This ensured that trap position remained exactly the same over the sampling period and disturbance reduced to minimum. Thus, we collected 25 samples (5 pitfall traps × 5 sampling dates) for each site, for a total of 2,250 samples. This spatially intensive sampling allowed us to obtain extremely detailed data about species distribution at fine scale. Further details about study area and data collection can be found in Tsafack et al. (2019b, 2020a). For each site, we pooled the catches by period and calculated species abundance as the average among the five sampling dates. Carabids were identified to species based on keys and museum specimens with the aid of a taxonomist expert in carabid beetles (Prof. H. Liang, see Acknowledgments). Voucher specimens are preserved in the collections of the School of Agriculture, Ningxia University.

### Data analysis

We calculated the Pianka index (Pianka 1973) of niche overlap to express spatial overlap between species pairs, using EcoSimR 1.00 (Gotelli et al. 2015):

$O_{jk}=O_{kj}=\frac{\sum_{i}^{n}p_{ij}p_{ik}}{\sqrt{\sum_{i}^{n}p_{ij}^{2}\sum_{i}^{n}p_{ik}^{2}}}$

where *O_jk_* is Pianka’s index of niche overlap between species *j* and *k*, *p_ij_* is the proportion of the *i*th resource used by species *j*, *p_ik_* is the proportion of the *i*th resource used by species *k*, and *n* is the total number of resources. In our case, the resource is the habitat space (sampling sites), the use of which is assumed to be expressed by species abundances. The *O_jk_* index is a symmetrical modification of the asymmetric index proposed by MacArthur and Levins (1967) for estimating competition coefficients from field data about resource utilization. It is an analogue of a correlation coefficient and ranges from zero (complete dissimilarity in resource utilization between two species) to one (complete identity in resource utilization). Pianka’s symmetric measure is more convenient than the original MacArthur and Levins’ asymmetric form (May 1974). Moreover, there is a general agreement that overlap measures cannot be used as true competition coefficients (Hurlbert 1978; Abrams 1980; Holt 1987) because there is an inverse relationship between competition and niche overlap (Pianka 1976, 1978).

Following widespread criticism of quantifying competitive interactions based on overlap indices (e.g., Connell 1980), more modern analyses of niche overlap are meant as description of interspecific interactions *sensu lato*, unless competitive interactions are known to occur *a priori* (Holt 2009; Lombardo and Mjelde 2014). Thus, following the current views in ecology, we have not automatically implied competition from *O_jk_* values, but used them merely as measures of niche overlap (see Colwell and Futuyma 1971; Pianka 1974 for the distinction between overlap and competition). However, negative (competitive-like) mutual influences of two coexisting species may be suspected when overlap over limited key resources is high (Chesson 2011) but, on the long term, competitive exclusion should lead to low niche overlap. Co-existence does not imply competition, but we believe that our analyses can provide useful insights into potential competition processes.

To assess if the mean values of niche overlap differed from those expected by chance, we compared the observed average values of spatial niche overlap (i.e., the averages of the niche overlap values between each species pair) with the expected averages obtained from simulated null-assemblages (pseudo-communities) constructed with two alterative sets of rules (see below). In each case, we calculated 10,000 null-assemblages, a number of permutations considered enough to avoid algorithm biases in calculations (Lehsten and Harmand 2006).

Null-assemblages were simulated using Monte Carlo randomization algorithms that assign resource use values (in our case, number of individuals from different sampling sites) to each species. The choice of an appropriate model to construct null-assemblages is a critical issue. Lawlor (1980) developed four randomization algorithms that differ in whether utilizations are reshuffled or replaced by a random number, and in whether the zeros in the matrix are retained or not. These algorithms are referred to as RA1, RA2, RA3, and RA4. Both retaining/relaxing niche breadths and retaining/reshuffling zeroes have implications for the structure of the null-assemblage and affect the power of the test (Gotelli and Graves 1996). Theoretical and empirical analyses of these algorithms (Winemiller and Pianka 1990; Kobayashi 1991) led to the conclusion that Lawlor’s (1980) RA3 is the best existing algorithm for use in null models of resource overlap. This algorithm tests for community structure by retaining niche breadth (i.e., the amount of specialization) for each species through simulated specialization equal to the observed value, but reshuffles zero states (i.e., by randomly varying the particular resources that were used, in our case space utilization). This destroys any guild structure resulting strictly from the zero structure of the resource utilization matrix.

The RA3 algorithm relies on the equiprobability assumption that each species will use the resource in a site with the same probability or each site will have the same probability to host a species (in other words, differences between rows (species) or columns (sites) are not preserved). The RA3 algorithm tends to overestimate niche overlap if the equiprobability assumption is not met, because more abundant resources will be used by all species even if niche segregation occurs. Thus, for comparative purposes, we also used the RA2 algorithm, which tests for structure in the generalist-specialist nature of the resource utilization matrix by conserving guild structure (zero states are retained, thus preventing species that did not use a certain resource in the field from doing so in simulations), but relaxes niche breadth (i.e., it allows niche breadth to vary, thus assuming a random equiprobable specialization) (Gotelli and Graves 1996). In all cases, equiprobable resource use was *a priori* assumed in the analyses.

### Species co-occurrence

We also calculated species segregation by using the C-score (Stone and Roberts 1990). The C-score for a species pair *jk* is calculated as:

*C_jk_*=(*R_j_*–*SS*)(*R_k_*–*SS*)

where *R_j_* is the row total for species *j*, *R_k_* is the row total for species *k*, and SS is the number of samples that contain both *j* and *k.* Thus, for any particular species pair, the C-score is a numerical index that ranges from a minimum of 0 (maximally aggregated) to a maximum of *R_j_R_k_* (maximally segregated with no shared samples). The matrix-wide C-score is an average of all the pairwise values of C-score for different species, so it reflects both positively and negatively associated species pairs. To establish whether the matrix had an average C-score significantly different from what can be expected from a null model, we compared the observed values with the expected averages obtained from 10,000 simulated null-assemblages using the fixed row-fixed column (FF) algorithm, which preserved row and column totals (Ulrich and Gotelli 2007; Strona et al. 2014).

While niche overlap analysis uses a data matrix with species- and site-specific observed abundances, the C-score searches for non-random structure in the species assemblage data by using a presence/absence data matrix (Gotelli and Graves 1996). High average C-scores indicates a low randomness, i.e., a greater likelihood that the distribution of one species has been directly affected by the presence of other species. Thus, for an assemblage that is competitively structured, the C-score should be significantly larger than a randomly assembled community (Gotelli 2000).

## Results

We collected a total of 25 species of carabid beetles (Table 1). *Amara helva* Tschitscherine, 1898, *Corsyra fusula* (Fischer Von Waldheim, 1820), *Cymindis binotata* Fischer Von Waldheim, 1820, and *Harpalus lumbaris* Mannerheim, 1825 were present only in the desert steppe (Table 1). *Carabus vladimirskyi* Dejean, 1830 was present in all types of steppe. *Carabus vladimirskyi* was present in all sites in the two first sectors of the typical steppe but in only six sites in the third typical steppe. *Carabus vladimirskyi* was present in 15 and 10 sites in the first and second sector of the meadow steppe, respectively. In the desert steppe, *C. vladimirskyi* was present only in one site.

**Table 1.** Species distribution of carabid beetles in three types of Central Asian steppes. Number of sites (N. sites) in which each species was found and number of collected individuals (N. ind.) are also given.

	Desert steppe		Typical steppe 1		Typical steppe 2		Typical steppe 3		Meadow steppe 1		Meadow steppe 2	
	N. sites	N. ind.	N. sites	N. ind.	N. sites	N. ind.	N. sites	N. ind.	N. sites	N. ind.	N. sites	N. ind.
*Amara dux* Tschitscherine, 1894	3	6	8	11	6	10	9	31	0	0	6	9
*Amara harpaloides* Dejean, 1828	1	7	0	0	3	3	0	0	0	0	1	1
*Amara helva* Tschitscherine, 1898	3	9	0	0	0	0	0	0	0	0	0	0
*Amara* sp.	0	0	2	3	4	6	0	0	1	1	4	5
*Broscus kozlovi* Kryzhanovskij, 1995	0	0	1	2	0	0	0	0	1	1	3	5
*Calosoma anthrax* Semenov, 1900	0	0	9	17	7	11	5	6	0	0	5	7
*Calosoma chinense* Kirby, 1819	1	1	1	1	0	0	0	0	0	0	1	1
*Calosoma lugens* Chaudoir, 1869	0	0	4	6	2	3	0	0	0	0	2	2
*Carabus anchocephalus* Reitter, 1896	0	0	1	1	3	3	11	25	0	0	14	56
*Carabus crassesculptus* Kraatz, 1881	0	0	0	0	0	0	0	0	15	295	11	44
*Carabus gigoloides* Cavazzuti, 2000	0	0	0	0	0	0	0	0	14	266	1	1
*Carabus glyptoterus* Fischer Von Waldheim, 1827	15	252	14	66	14	194	15	327	0	0	14	47
*Carabus modestulus* Semenov, 1887	0	0	0	0	0	0	0	0	15	82	1	2
*Carabus sculptipennis* Chaudoir, 1877	0	0	15	247	14	140	6	14	2	2	1	1
*Carabus vladimirskyi* Dejean, 1830	1	3	15	560	15	639	6	13	15	808	10	16
*Corsyra fusula* (Fischer Von Waldheim, 1820)	1	3	0	0	0	0	0	0	0	0	0	0
*Cymindis binotata* Fischer Von Waldheim, 1820	7	19	0	0	0	0	0	0	0	0	0	0
*Dolichus halensis* (Schaller, 1783)	0	0	0	0	1	2	1	1	0	0	0	0
*Harpalus lumbaris* Mannerheim, 1825	4	11	0	0	0	0	0	0	0	0	0	0
*Poecilus fortipes* (Chaudoir, 1850)	0	0	15	227	14	103	6	8	13	120	15	94
*Poecilus gebleri* (Dejean, 1828)	0	0	15	391	15	608	15	135	2	2	7	9
*Pseudotaphoxenus mongolicus* (Jedlicka, 1953)	11	23	3	4	10	39	4	11	0	0	0	0
*Pseudotaphoxenus rugupennis* (Faldermann, 1836)	3	3	15	85	15	133	15	39	6	11	12	39
*Reflexisphodrus reflexipennis* (Semenov, 1889)	0	0	1	2	0	0	0	0	14	332	10	34
*Zabrus potanini* Semenov, 1889	1	1	10	25	11	54	0	0	7	16	10	20

**Figure 1.**
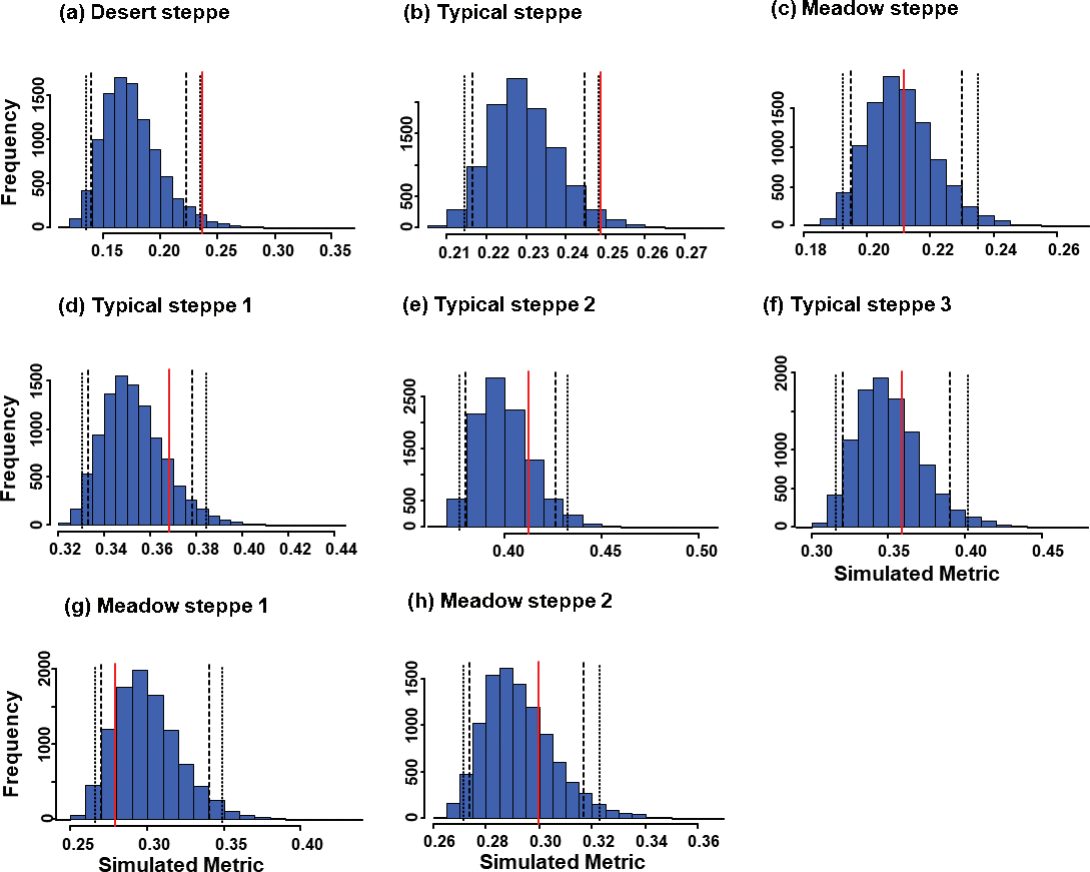
Histograms of expected values (blue bars) for niche overlap in carabid beetle communities of Central Asian steppes using the RA3 algorithm to generate 10,000 null matrices. Investigated ecosystems were a desert steppe (**a**), a typical steppe (**b**), a meadow steppe (**c**), three sectors within the typical steppe (**d–f**), and two sectors within the meadow steppe (**g, h**). In each graph, the vertical red line indicates the observed value, long-dash lines indicate the one-tailed 95% limits, and the short-dash lines the two-tailed 95% limits.

**Figure 2.**
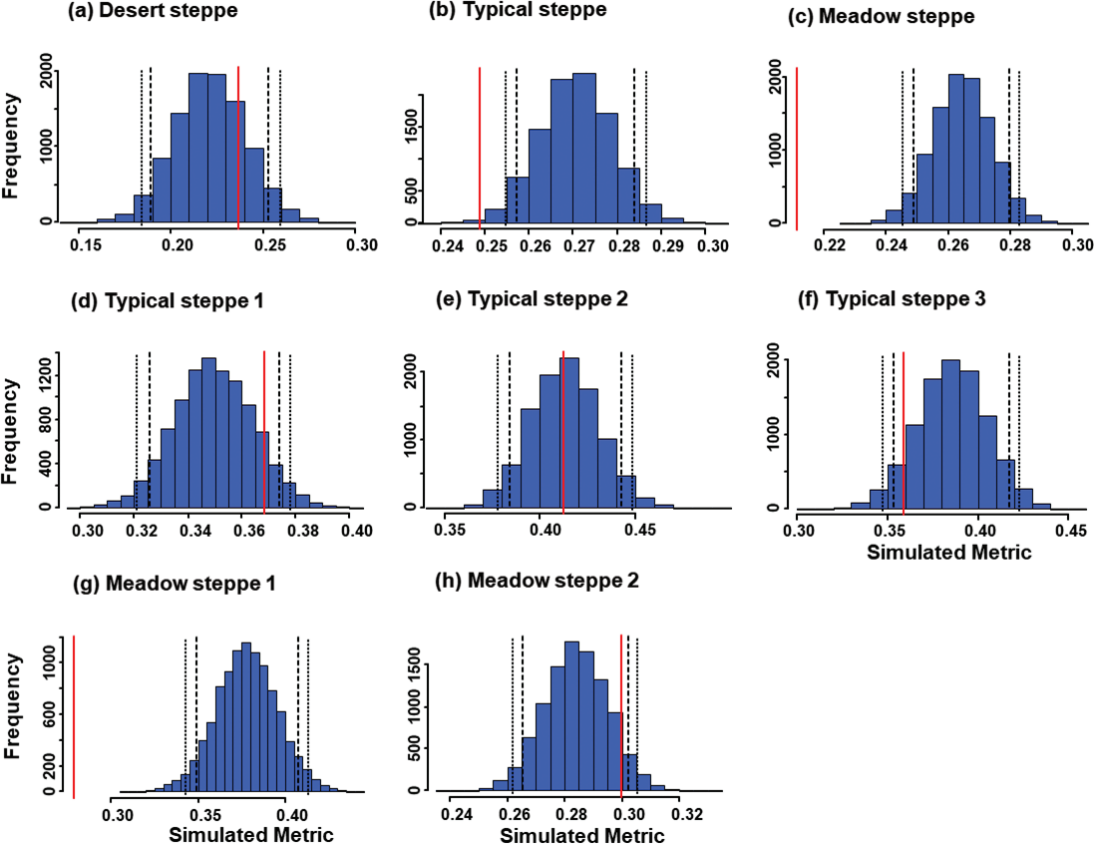
Histograms of expected values (blue bars) for niche overlap in carabid beetle communities of Central Asian steppes using the RA2 algorithm to generate 10,000 null matrices. Investigated ecosystems were a desert steppe (**a**), a typical steppe (**b**), a meadow steppe (**c**), three sectors within the typical steppe (**d–f**), and two sectors within the meadow steppe (**g, h**). In each graph, the vertical red line indicates the observed value, long-dash lines indicate the one-tailed 95% limits, and the short-dash lines the two-tailed 95% limits.

Overall mean pairwise niche overlap was 24% in the desert steppe, 25% in the typical steppe and 21% in the meadow steppe when these ecosystems were considered as a whole (Table 2). When the sectors were analyzed separately, values of mean niche overlap were 37%, 41% and 36% in the three sectors of the typical steppe, whereas in the two sectors of the meadow steppe they were 28% and 30%, respectively. Mean overall niche overlap in the desert was significantly higher than the respective simulated assemblages using the RA3 algorithm (P = 0.02; Table 2, Fig. 1). This community also had the highest value of variance among all those investigated in this study. The desert steppe did not vary significantly from randomness using the RA2 algorithm (Table 2, Fig. 2).

**Table 2.** Results of null-model species-niche overlap for desert, typical, and meadow steppes: Pianka index estimates and variances of observed values and expected values. Expected values are obtained with RA3 and RA2 algorithms. Significant values are in bold.

Steppe	Observed		Expected (RA3) (10,000 iterations)				Expected (RA2) (10,000 iterations)			
	estimate	variance	estimate	variance	Lower-tail	Upper-tail	estimate	variance	Lower-tail	Upper-tail
					P = (Obs < exp)	P = (Obs > exp)			P = (Obs < exp)	P = (Obs > exp)
Desert steppe	0.24	0.081	0.17	<0.001	0.9	**<0.05**	0.22	<0.001	0.8	0.2
Typical steppe	0.25	0.048	0.23	<0.001	0.9	**<0.05**	0.27	<0.001	**<0.01**	0.9
Meadow steppe	0.21	0.046	0.21	<0.001	0.6	0.4	0.26	<0.001	**<0.001**	1.0
Typical steppe 1	0.37	0.080	0.35	<0.001	0.9	0.1	0.35	<0.001	0.9	0.1
Typical steppe 2	0.41	0.058	0.40	<0.001	0.8	0.1	0.41	<0.001	0.5	0.5
Typical steppe 3	0.36	0.051	0.35	<0.001	0.7	0.3	0.39	<0.001	0.1	0.9
Meadow steppe 1	0.28	0.055	0.30	<0.001	0.2	0.8	0.38	<0.001	**<0.00001**	1.0
Meadow steppe 2	0.30	0.063	0.29	<0.001	0.7	0.3	0.28	<0.001	0.9	0.8

Mean overall niche overlap in the typical steppe was also significantly higher than in the simulated assemblages constructed using the RA3 randomization algorithm (P = 0.02; Table 2, Fig. 1). However, using the RA2 algorithm (Table 2, Fig. 2), the mean overall niche overlap in the typical steppe was significantly lower than in the simulated assemblages (P < 0.01). The mean niche overlap of carabid species in three sectors of the typical steppe did not differ significantly from the expected regardless of the randomization algorithm used (P > 0.05 in all cases; Table 2, Fig. 2).

The mean niche overlap in the meadow steppe was significantly lower than expected under RA2 (P < 0.001), but not under RA3 (P = 0.444) (Table 2, Figs 1, 2). When the two sectors were analyzed separately, mean overall niche overlap for the first sector was significantly lower than expected under the RA2 randomization algorithm (P < 0.001), but not under the RA3 algorithm (Figs 1, 2).

**Figure 3.**
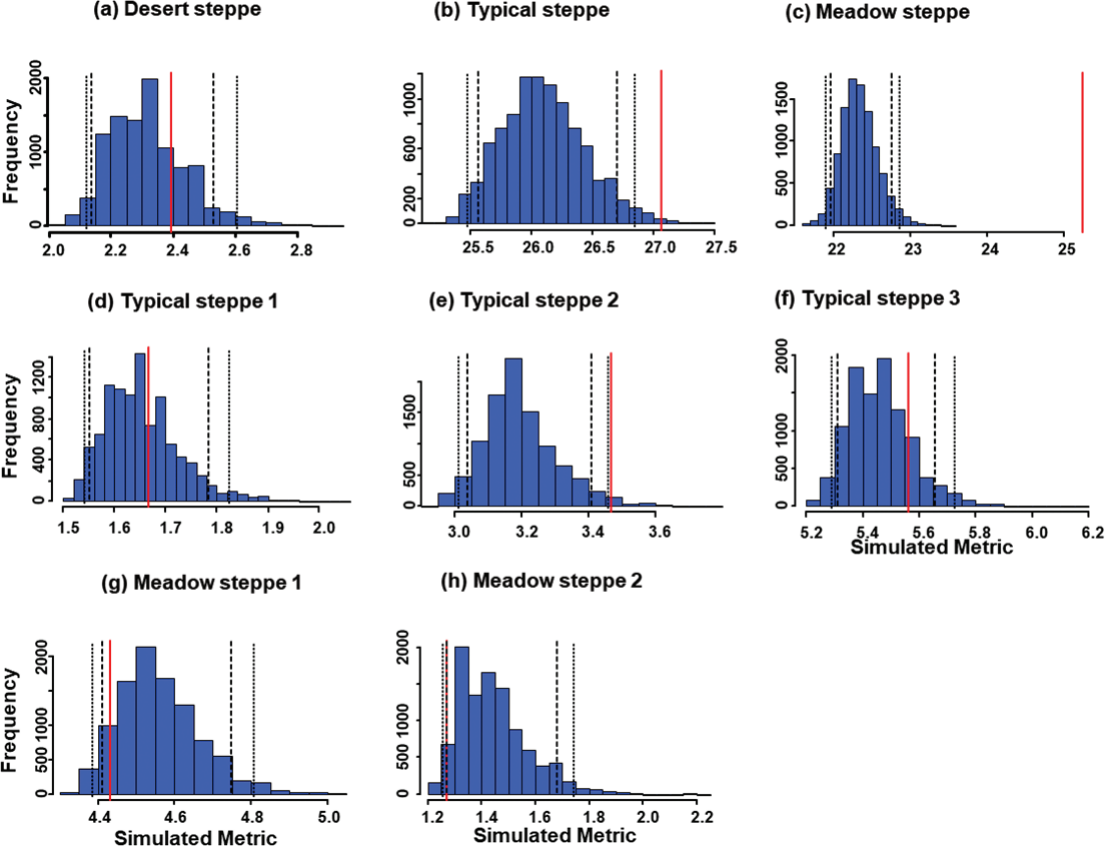
Histograms of expected values (blue bars) for species co-occurrence (c-scores) in carabid beetle communities of Central Asian steppes using the fixed-fixed algorithm to generate 10,000 null matrices. Investigated ecosystems were a desert steppe (**a**), a typical steppe (**b**), a meadow steppe (**c**), three sectors within the typical steppe (**d–f**), and two sectors within the meadow steppe (**g, h**). In each graph, the vertical red line indicates the observed value, long-dash lines indicate the one-tailed 95% limits, and the short-dash lines the two-tailed 95% limits.

Expected C-scores were very large in the typical and meadow steppe; however, they were low with high variance in the desert steppe (Table 3, Fig. 3). C-scores revealed that increases in niche overlap were paralleled by reductions in species segregation. Analysis of co-occurrences showed that species distribution in the desert steppe did not deviate significantly from a random pattern, but observed C-scores where higher than the mean of simulated values in both the typical and the meadow steppes (P < 0.01, Table 3, Fig. 3). However, when the three sectors of the typical steppe and the two sectors of the meadow steppe were analyzed separately, C-scores deviated significantly from randomness only in one sector of the typical steppe (Table 3, Fig. 3).

**Table 3.** Results of null-model co-cooccurrence species for desert, typical, and meadow steppes: c-score estimates and variances of observed values and expected values. Expected values are obtained with Sim9 (fixed-fixed) algorithm. Significant values are in bold.

Steppe	Observed		expected (Sim9 (Row Sums = Fixed; Col Sums = Fixed)) (10,000 iterations)			
	estimate	variance	estimate	variance	Lower-tail	Upper-tail
					P = (Obs < exp)	P = (Obs > exp)
Desert steppe	2.40	11.596	2.32	2.318	0.8	0.3
Typical steppe	27.07	2066.180	26.10	0.119	0.9	**<0.01**
Meadow steppe	25.24	1247.348	22.34	0.059	1.0	**<0.0001**
Typical steppe 1	1.67	14.980	1.65	0.005	0.6	0.4
Typical steppe 2	3.47	28.867	3.20	0.013	0.9	**<0.05**
Typical steppe 3	5.56	59.436	5.46	0.013	0.8	0.2
Meadow steppe 1	1.27	8.448	4.56	0.012	0.1	0.9
Meadow steppe 2	4.43	32.235	1.44	0.017	0.1	0.9

## Discussion

### The role of competition in carabid beetles: an open question

The role of competition in carabid beetles is debated. Interspecific competition in carabid beetle communities was reviewed in detail by Niemelä (1993), who found support for interspecific competition in carabid communities or evidence of resource partitioning in only half of the studies examined.

Laboratory studies have produced contrasting results. For example, laboratory research showed that mortality rates observed when two species of *Pterostichus* were reared together did not differ from those observed when they were reared separately (Paarmann 1966). However, other research found that, when reared separately, different species of the genus *Scarites* can show low mortality, but if they are reared together, they show extremely high aggressive behaviors even at low density (Peyrieras 1976). Experimental laboratory studies involving *Pterostichus adstrictus* Eschscholtz, 1823, and *P. melanarius* (Illiger, 1798) indicated that intraguild predation and interspecific competition for food among both larvae and adults have the potential to reduce survival and reproductive potential in nature (Currie et al. 1996). Nonetheless, such effects could not be documented in a two-year field experiment (Niemelä et al. 1997).

Field studies have produced contradictory results. Field research performed by den Boer (1979) showed population stability through years in co-occurring congeneric species, a result that contrasts with the idea that competition plays a major role in defining their distribution. These results were obtained by comparing species abundances of adult beetles found in pitfall traps kept at the same places over years. However, it is possible that adults do not compete at all, but that competition structures communities through larval competition (see, for example, Baars and Van Djik 1984). It has been also suggested that species that seem to co-occur might in fact occupy different microhabitats (Abrams 1986; Zalewski et al. 2014). In a study of two syntopic species of the genus *Carabus*, Lenski (1982) showed that *C. sylvosus* Say, 1823 attained a larger average body size where *C. limbatus*, 1825 was less abundant, suggesting the influence of competition (Lenski 1982). Studies based on serological (Sergeeva 1994) and isotopic approaches (Zalewski et al. 2014) have documented high niche overlap in species diets, which indicates that food does not constitute a primary factor of competition between some co-occurring carabid species.

It has been suggested that carabid species with overlapping trophic niches can co-occur if they have different circadian rhythms. For example, *Notiophilus biguttatus* (Fabricius, 1779) and *Nebria brevicollis* (Fabricius, 1792) not only have their activity peaks in different seasons (in spring and in autumn, respectively), but also show different circadian patterns, being diurnal and nocturnal, respectively (Dennison and Hodkinson 1983). Most species in a Norwegian carabid assemblage occupied different biotopes or differed in body size, and the only two species that occupied the same biotopes and had the same size showed different seasonal activity patterns (Erikstad et al. 1989). Pearson and Mury (1979) studied potential niche separation in an assemblage of *Cicindela* species. They showed that prairie-dwelling species had mandibles of different sizes, suggesting that they feed on animals of different sizes. However, they explained overlap in mandible sizes of species living near pond edges, by suggesting that competition was low there because of higher local abundance of animals that prey on these tiger beetles keeping their populations low relative to resource supply.

Based on the high frequency of co-occurrence of congeneric species, some authors (Darlington 1971; Basilewski 1984; den Boer 1986) have argued that competition is not a driver of community structure in carabid beetles. However, other studies have shown that congeneric species do not tend to co-occur, and that co-occurring species tend to differ in size, occupy different habitats, or have different mating periods (Peyrieras 1976; Brandl and Topp 1985; Loreau 1986). Character displacement has been described in *Amblystogenium pacificum* (Putzeys, 1869) and *A. minimum* Luff, 1972 (Davies 1987). These two species have very different body size where they co-occur, but where *A. pacificum* is alone its size is more similar to that of *A. minimum* (Davies 1987). A similar situation has been recorded in cave-dwelling species communities (Van Zant et al. 1978). In this paper, we have broadened the discussion to investigate potential competition in carabid communities by using null model approaches to test deviation of species coexistence patterns from randomness.

### Insights from null model approaches

Niche complementarities (i.e., niche differences between species) have long been considered to be important key drivers of species coexistence (e.g. Darwin 1859; Macarthur and Levins 1967; Silvertown 2004; Stubbs and Wilson 2004; Mason and Wilson 2006). Nonetheless, local environmental conditions may blunt their effects, producing patterns in which co-occurring species are more similar than expected by chance (Leibold 1998; Mayfield and Levine 2010). Thus, niche similarities can be also observed, when the focal species are not regularly distributed in a niche space, but aggregated in groups of ecologically similar species, e.g., when resource limitation is not regularly a feature of the environment. Understanding how species sort themselves into communities is essential to understand how species co-occur to maintain biodiversity. Thus, important insights may come from observation and study of non-random patterns in community assembly. Such patterns do not necessarily depend on competition (Zalewski et al. 2014), but on the other hand they are not a simple product of chance and may depend on resource partitioning developed over evolutionary time frames (Behangana and Luiselli 2008). Use of null models helps us to discover non-random patterns and to link them to underlying mechanisms through further study. In fact, our null model approaches, in which the resource is the space designated by capture in a particular site, are useful to suggest potential species interactions for more direct tests. For example, species that are collected in the same place might not interact if they are active in different moments of the day. Yet, comparison of null models constructed by using different rules may disclose patterns that can be used to develop hypotheses about mechanisms. In particular, our use in this study of two different null models to understand the spatial distribution of carabid beetles provided complementary information.

### Patterns in the desert and typical steppes

The RA3 algorithm, which is based on the assumption of retaining niche breadth, revealed that the spatial niches of carabid species overlapped significantly and more than expected in both the desert and the typical steppe. These results were obtained when niche breadths were preserved but species were allowed to be modelled as present in sites where they were not found in the observed data set. This indicates that some places are unsuitable for particular species, and that existence of such places likely forces spatial niche overlap in places that are suitable. This result is consistent with the idea that in these ecosystems resources are scarce and structured spatially, so that unsuitable parts of the environment (here, for example, sandy areas without vegetation) may restrict species from finding areas of suitable habitat. This result seems to reflect that mobility of carabids is strongly constrained in desert steppe, as we found in a previous paper (Tsafack et al. 2020b). In that work, we demonstrated that there were significant correlations between carabid activity-density and vegetation indices at a small scale (300 m) in desert steppe, although they disappeared at large scales (buffers zones of 1400–1500 m diameter; Tsafack et al. 2020b). Furthermore, the significant spatial niche overlap observed in the desert steppe is likely related to the low community stability found in this ecosystem (Tsafack et al. 2019a). This instability may produce substantial temporal changes in species composition of local assemblages, which interferes with the evolution of competitive interactions. In contrast, in the typical steppe, spatial niche overlap was significant, but community stability was higher and did not differ from that in meadow steppe (Tsafack et al. 2019a). This result suggests that niche overlap can be high even when communities are stable, if the habitat (i.e., extent of vegetation cover) is homogeneous.

Our analysis was strictly about spatial distribution, and we did not consider explicitly the distribution of vegetation or productivity. However, the desert steppe supports low vegetative productivity that is not uniformly distributed, so we think that spatial segregation patterns likely reflect uneven resource distribution. Thus, that the species aggregation observed in our data is driven by beetle concentration in places where resources are more abundant. This is in agreement with den Boer’s (1986) “coexistence principle”, which states that ecologically closely related species frequently coexist in the same habitats, if interspecific interference is not more important than intraspecific competition.

The carabids of the desert community also showed the highest variance in niche values, i.e., some species pairs show high niche overlap and others show low niche overlap. Thus, we argue that spatial resource partitioning is due to the presence of sites that are favorable to certain species but not to others, which supports our prediction H1. The assumption of resource partitioning can also be supported by the high variance observed in carabid functional diversity in desert steppe (Tsafack et al. 2019b), which suggests that species are associated with different microhabitat or use different resources.

### Patterns in the meadow steppe

The carabid community of the meadow steppe did not deviate significantly from randomness under the RA3 assumptions, which means that the environment is used in a more uniform way, with no or few spaces unavailable to carabids. This is consistent with the greater landscape homogeneity of this ecosystem as postulated by our prediction H2. A previous study showed that carabid activity-density in the meadow steppe was significantly correlated with vegetation indices at a relatively large scale (buffers zones of 1450 m and 1500 m diameter), thus suggesting that carabid species in this ecosystem are more mobile and can exploit larger areas of suitable habitat (Tsafack et al. 2020b). Thus, differences in dispersal ability are consistent with observed differences in species co-occurrences (Zalewski et al. 2018).

### The effect of the zero-structure

Modelled with the RA2 algorithm (which retains the zero-structure of the matrix, but allows niche breadths to vary), niche overlaps in the beetle assemblage of the desert steppe did not vary significantly from randomness. This indicates that some places are unavailable to some species, but there are no other constraints on space utilization. Although the carabids of this ecosystem divide the shared spatial resource in a random way, some places are apparently unsuitable for particular species. For data from the typical steppe, however, niche overlap values from the RA2 algorithm were lower than expected if determined by chance, suggesting that the environment is spatially structured. In other words, presence of zeroes in the matrix of habitat use are important, and if they are removed niche overlap will increase significantly. Thus, the unsuitability of some sites for particular species contributes to the pattern of spatial utilization among species. Similarly, in the meadow steppe, use of the RA2 algorithm leads to lower niche overlap than expected by chance; however, if the influence of zeroes is removed (RA3), niche overlap does not increase to become significantly higher than by chance. This suggests that places unavailable to particular species are less important to assemblage structure in the meadow steppe than in the other two ecosystems. This interpretation should be offered with caution however, because use of RA2 algorithm is prone to Type I error (false positive) (Gotelli et al. 2015).

### Within-ecosystem analyses

If the three sectors of the typical steppe are analyzed separately, neither the RA2 nor RA3 approach revealed non-random community structure. This suggests that spatial structure in the overall analysis arose because the three sectors include different assemblages. This is not surprising as the abundance of even the most widely distributed species (*C. vladimirskyi*) varied widely in this ecosystem, being trapped commonly in two sectors, but rarely in the third. Thus, spatial niche separation likely results from low overlap in patterns of habitat use among these three assemblages more than spatial segregation within each sector. In other words, spatial segregation was greater between species found in different sectors than between species within the same sector. This is an interesting result showing that spatial niche overlap values should be interpreted with consideration of the scale of analysis. At a broad scale, niche separation appears to result from species segregation reflecting their different distributions, but when the analysis is conducted at a finer scale, species appeared to be no more segregated than expected by chance. These results are consistent with Loreau’s (1986) results about forest carabids, where there was no spatial separation of species because of habitat homogeneity.

The situation is paralleled in the meadow steppe in the second sector, but although overall niche overlap in the first sector was lower than expected. This suggests that the first sector has a high habitat heterogeneity, that facilitates species segregation. This is consistent with the strong correlations that we found between carabid activity density and vegetation indices only at a larger scale (buffers of 1450 m and 1500 m diameters; Tsafack et al. 2020b).

### What patterns of co-existence tell us about competition?

Species that coexist in a certain area are expected to show lower overlap than a randomly assembled set of species (from the same area) if the community is structured by competition because of mutual exclusion. The significant overlap in spatial distribution found for the carabids of the desert and typical steppes using the RA3 algorithm thus suggests that there is little competition (Pianka 1986; Gotelli and Graves 1996), or perhaps strong competition that did not lead to a divergence on the use of space until the present (Gotelli and Graves 1996). As niche overlap was higher than random in the desert and the typical steppes, but not in the meadow steppe, with the RA3 but not with the RA2 algorithm, we believe that the spatial fragmentation of resource distributions causes some degree of niche overlap.

The high average C-scores found in the meadow and typical steppe suggests that the presence of a species is directly affected by the presence of other species, and hence that competition may have some role in defining species assemblages in these ecosystems, whereas the low value observed in the desert suggests, as above, that competition is not involved here.

## Conclusions

Our analyses based on the use of null models constructed under different sets of assumptions reveal that spatial niche overlap values in carabid communities inhabiting Central Asian steppes reflect both habitat structure and species interactions, and that results are scale dependent. Niche overlaps were significantly higher than expected by chance alone in the desert steppe, where resources are highly fragmented, and therefore species tend to be aggregate and to share resources. In the typical and meadow steppes, at a broad scale, we found species segregation, but when the analysis was conducted at a finer scale, species appeared to be not more segregated than expected. This indicates that, in homogenous landscapes, species are not segregated by the habitats and tend to co-occur more randomly. However, high average co-occurrence found in the meadow and typical steppes indicates that the distribution of one species may be negatively affected by the presence of other species, and hence that competition may have some role in defining species assemblages in these ecosystems. By contrast, the very low co-occurrence value observed in the desert suggests that competition cannot be involved there. Thus, in homogeneous landscapes competition may play some role in community structure and biodiversity maintenance, whereas species assemblages are more driven by the spatial distribution of resources in a landscape where they are more fragmented.

## References

[B1] Abrams P (1980) Some comments on measuring niche overlap. Ecology 61: 44–49. 10.2307/1937153

[B2] Abrams PA (1986) Character displacement and niche shift analyzed using consumer-resource models of competition. Theoretical Population Biology 29: 107–160. 10.1016/0040-5809(86)90007-9 3961709

[B3] Baars MA Van Djik TS (1984) Population dynamics of two carabid beetles at a Dutch Heathland: I. Subpopulation fluctuations in relation to weather and dispersal. Journal of Animal Ecology 53: 375–388. 10.2307/4522

[B4] Basilewski P (1984) Essai d’une classification supragénérique naturelle des carabides lébiens d’Afrique et de Madagascar ColeopteraCarabidaeLebiinae). Revue de Zoologie Africaine 98: 525–559.

[B5] Behangana M Luiselli L (2008) Habitat niche community-level analysis of an amphibian assemblage at Lake Nabugabo, Uganda. Web Ecology 8: 125–134. 10.5194/we-8-125-2008

[B6] den Boer PJ (1979) Exclusion or coexistence and the taxonomic or ecological relationship between species. Netherlands Journal of Zoology 30: 278–306. 10.1163/002829679X00421

[B7] den Boer PJ (1986) The present status of the competitive exclusion principle. Trends in Ecology & Evolution 1: 25–28. 10.1016/0169-5347(86)90064-9 21227775

[B8] Brandl R Topp W (1985) Size Structure of *Pterostichus* spp. (Carabidae): Aspects of competition. Oikos 44: 234–238. 10.2307/3544694

[B9] Chase JM Leibold MA (2003) Ecological Niches: Linking Classical and Contemporary Approaches. University of Chicago Press, Chicago, 221 pp. 10.7208/chicago/9780226101811.001.0001

[B10] Chesson P (2011) Ecological niches and diversity maintenance, Research in Biodiversity. In: Pavlinov IYa (Ed.) Models and Applications.IntechOpen, 43–60. 10.5772/24534

[B11] Colwell RK Futuyma DJ (1971) On the measurement of niche breadth and overlap. Ecology 52: 567–576. 10.2307/1934144 28973805

[B12] Connell JH (1980) Diversity and the coevolution of competitors, or the ghost of competition past. Oikos 35: 131–138. 10.2307/3544421

[B13] Currie CR Spence JR Niemelä J (1996) Competition, cannibalism and intraguild predation among ground beetles (Coleoptera: Carabidae): A laboratory study. The Coleopterists Bulletin 50: 135–148.

[B14] Darlington PJ (1971) Carabidae on tropical islands especially the West Indies. In: Stern WL (Ed.) Adaptive Aspects of Insular Evolution. Washington State University Press, 85 pp.

[B15] Darwin C (1859) On the Origin of Species by Means of Natural Selection, or the Preservation of Favoured Races in the Struggle for Life. J. Murray, London, 466 pp. https://www.biodiversitylibrary.org/page/39563570 PMC5184128 30164232

[B16] Davies L (1987) Long adult life, low reproduction and competition in two sub-Antarctic carabid beetles. Ecological Entomology 12: 149–162. 10.1111/j.1365-2311.1987.tb00994.x

[B17] Dennison DF Hodkinson ID (1983) Structure of the predatory beetle community in a woodland soil ecosystem. I. Prey selection. Pedobiologia 25: 109–115.

[B18] Erikstad KE Byrkjedal I Kålås JA (1989) Resource partitioning among seven carabid species on Hardangervidda, southern Norway. Annales Zoologici Fennici 26: 113–120. https://www.jstor.org/stable/23736062

[B19] Fattorini S Bergamaschi D Mantoni C Acosta TRA Di Giulio A (2016) Niche partitioning in tenebrionid species (Coleoptera: Tenebrionidae) inhabiting Mediterranean coastal dunes. European journal of entomology 113: 462–468. 10.14411/eje.2016.060

[B20] Fattorini S Mantoni C Di Biase L Strona G Pace L Biondi M (2020) Elevational patterns of generic diversity in the tenebrionid beetles (ColeopteraTenebrionidae) of latium (Central Italy). Diversity 12: e47. 10.3390/d12020047

[B21] Giménez Gómez VC Verdú JR Gómez-Cifuentes A Vaz-de-Mello FZ Zurita GA (2018) Influence of land use on the trophic niche overlap of dung beetles in the semideciduous Atlantic forest of Argentina. Insect Conservation and Diversity 11: 554–564. 10.1111/icad.12299

[B22] Gotelli NJ (2000) Null model analysis of species co-occurrence patterns. Ecology 81: 2606–2621. 10.1890/0012-9658(2000)081[2606:NMAOSC]2.0.CO;2

[B23] Gotelli NJ Graves GR (1996) Null models in ecology. Smithsonian Institution Press, Washington, 388 pp.

[B24] Gotelli NJ Hart EM Ellison AM (2015) EcoSimR: Null model analysis for ecological data. R package. http://github.com/gotellilab/EcoSimR

[B25] Hairston NG Smith FE Slobodkin LB (1960) Community structure, population control, and competition. The American Naturalist 94: 421–425. 10.1086/282146

[B26] Holt RD (1987) On the relation between niche overlap and competition: The effect of incommensurable niche dimensions. Oikos 48: 110–114. 10.2307/3565696

[B27] Holt RD (2009) Bringing the Hutchinsonian niche into the 21^st^ century: Ecological and evolutionary perspectives. Proceedings of the National Academy of Sciences 106: 19659–19665. 10.1073/pnas.0905137106 PMC2780934 19903876

[B28] Hurlbert SH (1978) The Measurement of niche overlap and some relatives. Ecology 59: 67–77. 10.2307/1936632

[B29] Hutchinson GE (1957) Concluding remarks. Cold spring harbor symposia on quantitative biology 22: 415–427. 10.1101/SQB.1957.022.01.039

[B30] Kang L Han X Zhang Z Sun OJ (2007) Grassland ecosystems in China: review of current knowledge and research advancement. Philosophical transactions of the royal society B: Biological Sciences 362: 997–1008. 10.1098/rstb.2007.2029 PMC2435566 17317645

[B31] Kobayashi S (1991) Interspecific relations in forest floor coleopteron assemblages: Niche overlap and guild structure. Population Ecology 33: 345–360. 10.1007/BF02513559

[B32] Koutsidi M Moukas C Tzanatos E (2020) Trait-based life strategies, ecological niches, and niche overlap in the nekton of the data-poor Mediterranean Sea. Ecology and Evolution 10: 7129–7144. 10.1002/ece3.6414 32760517 PMC7391318

[B33] Lawlor LR (1980) Structure and stability in natural and randomly constructed competitive communities. The American Naturalist 116: 394–408. 10.1086/283634

[B34] Lehsten V Harmand P (2006) Null models for species co-occurrence patterns: assessing bias and minimum iteration number for the sequential swap. Ecography 29: 786–792. 10.1111/j.0906-7590.2006.04626.x

[B35] Leibold MA (1998) Similarity and local co-existence of species in regional biotas. Evolutionary Ecology 12: 95–110. 10.1023/A:1006511124428

[B36] Lenski RE (1982) Effects of forest cutting on two *carabus* species: evidence for competition for food. Ecology 63: 1211–1217. 10.2307/1938845

[B37] Liu J Zhao W Li F (2015) Effects of shrub presence and shrub species on ground beetle assemblages (Carabidae, Curculionidae and Tenebrionidae) in a sandy desert, northwestern China. Journal of Arid Land 7: 110–121. 10.1007/s40333-014-0040-6

[B38] Lombardo P Mjelde M (2014) Quantifying interspecific spatial overlap in aquatic macrophyte communities. Hydrobiologia 737: 25–43. 10.1007/s10750-013-1716-1

[B39] Loreau M (1986) Niche differentiation and community organisation in forest carabid beetles. In: Boer PJ den Luff ML Mossakowski D Weber F (Eds) Carabid Beetles: Their adaptions ad dynamics.Gustav Fischer, Stuttgart, 465–487.

[B40] Macarthur R Levins R (1967) The limiting similarity, convergence, and divergence of coexisting species. The American Naturalist 101: 377–385. 10.1086/282505

[B41] Mason NWH Wilson JB (2006) Mechanisms of species coexistence in a lawn community: mutual corroboration between two independent assembly rules. Community Ecology 7: 109–116. 10.1556/ComEc.7.2006.1.11

[B42] May RM (1974) On the theory of niche overlap. Theoretical population biology 5: 297–332. 10.1016/0040-5809(74)90055-0 4460250

[B43] Mayfield MM Levine JM (2010) Opposing effects of competitive exclusion on the phylogenetic structure of communities. Ecology Letters 13: 1085–1093. 10.1111/j.1461-0248.2010.01509.x 20576030

[B44] Niemelä J (1993) Interspecific competition in ground-beetle assemblages (Carabidae): What have we learned? Oikos 66: 325–335. 10.2307/3544821

[B45] Niemelä J Spence JR Cárcamo H (1997) Establishment and interactions of carabid populations: an experiment with native and introduced species. Ecography 20: 643–652. 10.1111/j.1600-0587.1997.tb00433.x

[B46] Paarmann W (1966) Vergleichende untersuchungen ueber die bindung zweier carabidenarten (*Pterostichus angustatus* Dft. und *Pterostichus oblongopunctatus* F.) an ihre verschiedenen Lebensraeume. Zeitschrift für wissenschaftliche Zoologie 174: 83–176.

[B47] Pascual-Rico R Sánchez-Zapata JA Navarro J Eguía S Anadón JD Botella F (2020) Ecological niche overlap between co-occurring native and exotic ungulates: insights for a conservation conflict. Biological Invasions 22: 2497–2508. 10.1007/s10530-020-02265-x

[B48] Pearson DL Mury EJ (1979) Character divergence and convergence among tiger beetles (Coleoptera: Cicindelidae). Ecology 60: 557–566. 10.2307/1936076

[B49] Peyrieras A (1976) Insects coléoptères CarabidaeScaritinae. Biologie, faune de Madagascar. Orstom-CNRS, Paris, 161 pp. https://horizon.documentation.ird.fr/exl-doc/pleins_textes/doc34-08/21577.pdf

[B50] Pianka ER (1973) The Structure of lizard communities. Annual Review of Ecology and Systematics 4: 53–74. 10.1146/annurev.es.04.110173.000413

[B51] Pianka ER (1974) Niche overlap and diffuse competition. Proceedings of the National Academy of Sciences 71: e2141. 10.1073/pnas.71.5.2141 PMC388403 4525324

[B52] Pianka ER (1976) Competition and niche theory. In: May M (Ed.) Theoretical Ecology: Principles and Applications.Blackwell Scientific Publication, Oxford, 114–141.

[B53] Pianka ER (1978) Evolutionary Ecology (2^nd^ ed.). Harper and Row, New York, 377 pp.

[B54] Pianka ER (1986) Ecology and Natural History of Desert Lizards. Princeton University Press, New Jersey, 208 pp. 10.1515/9781400886142

[B55] Price WP Denno RF Eubanks MD Finke DL Kaplan I (2011) Insect Ecology: Behavior, Populations and Communities. Cambridge University Press, Cambridge, 828 pp. 10.1017/CBO9780511975387

[B56] Schowalter TD (2011) Insect ecology. An ecosystem approach (3^rd^ ed.). Elsevier Academic Press, London, 650 pp.

[B57] Sergeeva TK (1994) Seasonal Dynamics of Interspecific Trophic Relations in a Carabid Beetle Assemblage. Carabid Beetles: Ecology and Evolution. Kluwer Academic Publishers, Dordrecht, 367–370. 10.1007/978-94-017-0968-2_55

[B58] Silvertown J (2004) Plant coexistence and the niche. Trends in Ecology & Evolution 19: 605–611. 10.1016/j.tree.2004.09.003

[B59] Soberón J Arroyo-Peña B (2017) Are fundamental niches larger than the realized? Testing a 50-year-old prediction by Hutchinson. PLoS ONE 12: e0175138. 10.1371/journal.pone.0175138 PMC5389801 28403170

[B60] Stone L Roberts A (1990) The checkerboard score and species distributions. Oecologia 85: 74–79. 10.1007/BF00317345 28310957

[B61] Strona G Nappo D Boccacci F Fattorini S San-Miguel-Ayanz J (2014) A fast and unbiased procedure to randomize ecological binary matrices with fixed row and column totals. Nature Communications 5: e4114. 10.1038/ncomms5114 24916345

[B62] Stubbs WJ Wilson JB (2004) Evidence for limiting similarity in a sand dune community. Journal of Ecology 92: 557–567. 10.1111/j.0022-0477.2004.00898.x

[B63] Tsafack N Xie Y Wang X Fattorini S (2020a) Influence of climate and local habitat characteristics on carabid beetle abundance and diversity in northern Chinese steppes. Insects 11: 1–19. 10.3390/insects11010019 PMC7023069 31878317

[B64] Tsafack N Di Biase L Xie Y Wang X Fattorini S (2019a) Carabid community stability is enhanced by carabid diversity but reduced by aridity in Chinese steppes. Acta Oecologica 99: e103450. 10.1016/j.actao.2019.103450

[B65] Tsafack N Fattorini S Benavides Frias C Xie Y Wang X Rebaudo F (2020b) Competing vegetation structure indices for estimating spatial constrains in carabid abundance patterns in chinese grasslands reveal complex scale and habitat patterns. Insects 11: e249. 10.3390/insects11040249 PMC7240609 32316087

[B66] Tsafack N Rebaudo F Wang H Nagy DD Xie Y Wang X Fattorini S (2019b) Carabid community structure in northern China grassland ecosystems: Effects of local habitat on species richness, species composition and functional diversity. PeerJ 6: e6197. 10.7717/peerj.6197 PMC6330033 30643684

[B67] Ulrich W Gotelli NJ (2007) Disentangling community patterns of nestedness and species co-occurrence. Oikos 116: 2053–2061. 10.1111/j.2007.0030-1299.16173.x

[B68] Van Zant T Poulson TL Kane TC (1978) Body-size differences in carabid cave beetles. The American Naturalist 112: 229–234. 10.1086/283263

[B69] Vandermeer JH (1972) Niche theory. Annual Review of Ecology and Systematics 3: 107–132. 10.1146/annurev.es.03.110172.000543

[B70] Winemiller KO Pianka ER (1990) Organization in natural assemblages of desert lizards and tropical fishes. Ecological Monographs 60: 27–55. 10.2307/1943025

[B71] Yang Y Hui C (2020) How competitive intransitivity and niche overlap affect spatial coexistence. Oikos 130: 260–273. 10.1111/oik.07735

[B72] Yu X-D Luo T-H Zhou H-Z (2004) Carabus (Coleoptera: Carabidae) assemblages of native forests and non-native plantations in Northern China. Entomologica Fennica 15: 129–137. 10.33338/ef.84217

[B73] Zalewski M Dudek D Tiunov AV Godeau J-F Okuzaki Y Ikeda H Sienkiewicz P Ulrich W (2014) High niche overlap in the stable isotope space of ground beetles. Annales Zoologici Fennici 51: 301–312. 10.5735/086.051.0302

[B74] Zalewski M Hajdamowicz I Stańska M Dudek-Godeau D Tykarski P Sienkiewicz P Ciurzycki W Ulrich W (2018) β-diversity decreases with increasing trophic rank in plant – arthropod food chains on lake islands. Scientific Reports 8: e17425. 10.1038/s41598-018-34768-y PMC6258687 30479354

[B75] Zhang X Zhao G Zhang X Li X Yu Z Liu Y Liang H (2017) Ground beetle (Coleoptera: Carabidae) diversity and body-size variation in four land use types in a mountainous area near Beijing, China. The Coleopterists Bulletin 71: 402–412. 10.1649/0010-065X-71.2.402

